# Morphological variations of fissures, lobes, and hilar pattern of the lung in a select South African sample

**DOI:** 10.1007/s00276-024-03497-5

**Published:** 2024-10-10

**Authors:** Refilwe Seleka, Megan Petersen, Kentse Sana Mpolokeng

**Affiliations:** Department of Human Biology, Faculty of Health Science, University of Cape Town, Observatory, 7925 South Africa

**Keywords:** Accessory fissures, Lung fissures, Lung lobes, Hilum pattern, Variations

## Abstract

**Introduction:**

The lungs are essential respiratory organs divided into lobes by the horizontal and oblique fissures. The hilum, located on the mediastinal surface of each lung, is where the bronchus, pulmonary veins, and pulmonary arteries enter and exit. This study aims to investigate and record the variations in the morphology of lung fissures, lobes, and hilar patterns observed in a South African sample.

**Methods and Materials:**

This cross-sectional observational study employed descriptive analysis. A total of 48 formalin-fixed bodies, comprising 24 females and 24 males from the Department of Human Biology at the University of Cape Town were studied were examined. Fissures were classified according to the Craig and Walker criteria.

**Results and Discussion:**

Incomplete oblique fissures were found in 25 right lungs and 30 left lungs. Incomplete horizontal fissures were observed in 39 right lungs, and one right lung exhibited the absence of both horizontal and oblique fissures. Accessory fissures were present in five right and seven left lungs. Variations in the hilar pattern were noted, including differences in the number and arrangement of structures in both right and left lungs.

**Conclusion:**

Variations were observed in the lobes, fissures and the hilar patterns of several lungs. Awareness of these morphological variations is crucial for surgeons and radiologist to avoid misdiagnosis and complications during surgical procedures.

## Introduction

The lungs, vital for respiration are positioned on either side of the mediastinum and are enclosed within their pleural sac [4]. The lungs are cone-shaped organs that consists of a concave base, an apex, three surfaces and three borders [1]. The base of each lung is positioned on the upper surface of the diaphragm, while the apex extends beyond the first rib [38]. The three surfaces of the lungs include: the costal surface lying next to the ribs and intercostal space of the thoracic wall; the mediastinal surface which lie in contact with the mediastinum anteriorly and the diaphragmatic surface which lie on the dome of the diaphragm [4, 41].The borders of the lungs include the inferior border, which separates the base of the lung from the costal surface, and the anterior and posterior borders which converge from the costal mediastinal surfaces [2]. The left lung is smaller and has a greater length compared to the right lung due to the dome of the diaphragm being higher on the right side, while the right lung has a greater width than the left lung due to the positioning of the heart extending towards the left side [37, 41].

The lungs are divided into lobes by fissures, such as the horizontal and oblique fissures, which are invaginations of visceral pleura. These fissures separate the lobes almost to the hilum, allowing for easy movement and uniform expansion of the lungs during respiration [14, 15]. The right lung is divided into three lobes, namely: the superior, middle, and inferior lobes separated by the horizontal and oblique fissures [24, 41]. The left lung on the other hand, is divided into two lobes, namely: the superior and inferior lobes, separated by an oblique fissure [7]. Additionally, the inferior border of the superior lobe of the left lung has a small tongue-like process called the lingula [26].

The mediastinal surface of the lung features a triangular depression known as the hilum, through which important structures enter and exit [15]. These structures include a principal bronchus, bronchial vessels, two pulmonary veins, a pulmonary artery, lymphatics, and nerves [4]. Both the right and left lung consists of two pulmonary veins and one pulmonary artery at the hilum, however, the bronchi differ in their subdivision between the right and left lung [36]. The right bronchus branches off into the superior lobe approximately 2.5 cm from the bifurcation of the trachea, with divisions above the pulmonary artery and is therefore termed the eparterial bronchus while other divisions will be below the pulmonary artery and thus termed the hyparterial bronchus [8, 29]. The left bronchus branches off below the pulmonary artery therefore named the hyparterial bronchus [8]. The structures at the hilum are arranged in different patterns between the right and left lung [15]. In the right lung, from superior to inferior, the structures are arranged as follows: eparterial bronchus, pulmonary artery, hyparterial bronchus and pulmonary veins, respectively [6]. In the left lung from superior to inferior, the structures are arranged as follows: pulmonary artery, hyparterial bronchus and pulmonary veins, respectively [29]. At both the lungs, from anterior to posterior, the structures are arranged as follows: pulmonary vein, pulmonary artery, and principal bronchus [6].

Any deviations from the normal embryological development will bring about variations in the morphology of the lobes and fissures as well as the hilum pattern [6, 26]. Knowledge of these variations, which may include absent, incomplete, and accessory fissures as well as variations in the number and arrangement of structures at the hilum is essential for planning different operative strategies for various surgical procedures such as thoracoscopic pulmonary resection or pulmonary lobectomy where a variation may cause complications [7].

Variability in lung fissure morphology is well-documented in the literature, with studies conducted in the United States of America, Turkey, Nepal, and Ethiopia [47]. However, only a few studies have been conducted on the African population. To date, only three case reports from Kenya, Nigeria and Tanzania, along with a cadaveric study conducted in Kenya have been published on this topic. Additionally, only one study by Mpolokeng et al. [27] has investigated anatomical variations in lung fissures within a South African sample. The morphology of the hilum is not well documented in the literature [36], and no paper has yet documented hilum anatomy in a South African sample. This study aims to address the variations in the morphology of lung fissures, lobes and hilar pattern observed in a South African sample.

## Methods and materials

A descriptive observational study was conducted at the Department of Human Biology Dissection halls, located in the Anatomy Building at the Faculty of Health Sciences, University of Cape Town. A total of 48 formalin-fixed bodies consisting of both females (*n* = 24) and males (*n* = 24) were examined. These bodies were those that had been previously dissected by medical and postgraduate honours students. The sample consisted of a total of 96 (48 left and 48 right) lungs. Poorly dissected lungs that lead to the destruction of lobes, fissures and the hilum structures were excluded from the study. Lungs that had severe pathologies were also excluded from the study. The lungs were analysed and observations were recorded on the data collection sheet. Data was recorded on the presence or absence of fissures and lobes, the completeness of fissures, the presence of accessory fissures, and the number and arrangement of structures at the hilum. Digital photographs were taken to further record the findings. The completeness of the lung fissures was analysed according to the Craig and Walker criteria, seen in Table 1.

**Table 1:** Grading of completeness of a fissures according to Craig and Walker criteria

Grades	Completeness of fissure
Grade I	Complete fissure with entire separate lobes
Grade II	Complete visceral cleft but parenchymal fusion at the base of the fissure
Grade III	Visceral cleft evident for part of the fissure
Grade IV	Complete fusion of the lobes with no evident fissural line

Permission to conduct the study was granted by the Cadaver Research Governance Committee: CRGC 2023/006. The study strictly adhered to the ethical guidelines and laws according to the Human Tissue Act of 2004 and the Declaration of Helsinki that pertain to the use of human cadaveric donors in anatomical research.

The statistical analysis for this study was conducted using the IBM SPSS Statistics version 28 software. For the categorical data, both Chi-squared test and frequency analysis were conducted. The Chi-squared test was also conducted to analyse the association between each pulmonary fissure, variations and sex.

## Results

### Anatomical variations of the lung fissures and lobes

A total of 96 lungs were examined, with 4 excluded. Of the 92 included lungs, the prevalence of variations in the oblique and horizontal fissures of 45 right lungs and 47 left lungs was observed and recorded according to the Craig and Walker criteria, as shown in Table 2.

**Table 2:** Prevalence of oblique, and horizontal fissure variations according to Craig and Walker criteria

Lungs	Fissures	Grade I *n* (%)	Grade II *n* (%)	Grade III *n* (%)	Grade IV *n* (%)
Right	Oblique	19 (42.2%)	7 (15.6%)	18 (40.0%)	1 (2.2%)
	Horizontal	5 (11.1%)	2 (4.4%)	37 (82.2%)	1 (2.2%)
Left	Oblique	17 (36.2%)	12 (25.5%)	18 (38.3%)	0

**Fig. 1:**
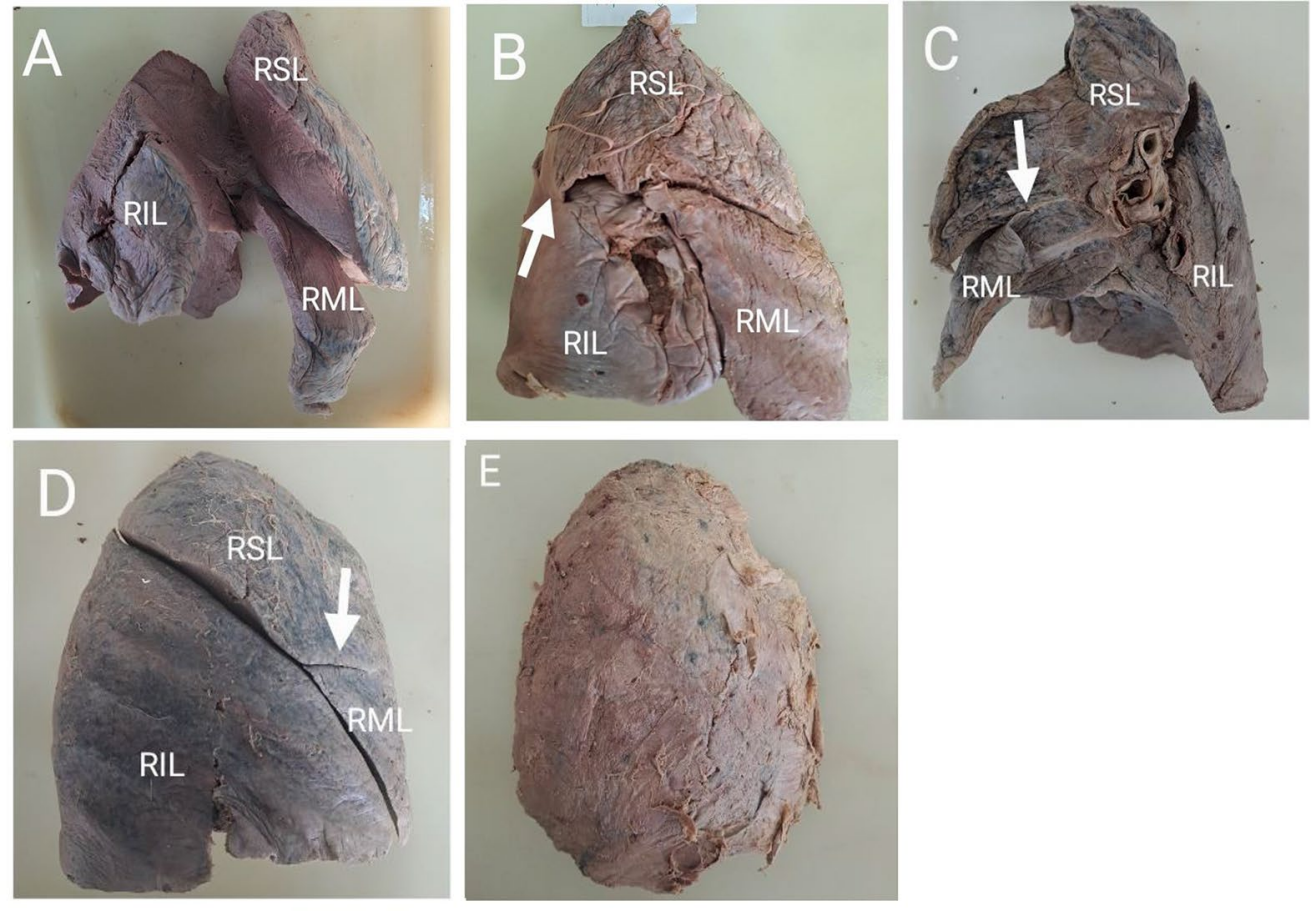
Morphological variations of the right lung. (**A**) Grade I oblique and horizontal fissure separating the RSL, RML and RIL completely. (**B**) Incomplete development of the oblique fissure causing the RSL tissue to be attached to RIL, as indicated by the white arrow. (**C**) Grade II horizontal fissure. The horizontal fissure is partially incomplete as it does not reach the hilum. White arrow indicates where it ends. (**D**) Grade II horizontal fissure, as indicated by the white arrow. (**E**) Grade IV oblique and horizontal fissures, forming one lobe. *Abbreviations* RSL=right superior lobe, RML= right middle lobe, RIL= right inferior lobe

Out of the 45 right lungs examined, 11.1% (*n* = 5/45) had Grade I horizontal fissures, 4.4% (*n* = 2/45) had Grade II, 82.2% (*n* = 37/45) had Grade III and 2.2% (*n* = 1/45) had Grade IV. For the oblique fissure, 42.2% (*n* = 19/45) had Grade I, 15.6% (*n* = 7/45) had Grade II, 40.0% (*n* = 18/45) had Grade III and 2.2% (*n* = 1/45) had Grade IV. No right lung was found to have only an absent horizontal fissure, nor was any right lung observed with solely an absent oblique fissure. However, both the horizontal and oblique fissure were absent in one lung (Fig. 1E). Among the 47 left lungs, 36.2% (*n* = 17/47) had Grade I oblique fissures, 25.5% (*n* = 12/47) had Grade II and 38.3% (*n* = 18/47) had Grade III oblique fissures. No Grade IV oblique fissure was seen in any of the left lungs.

**Fig. 2:**
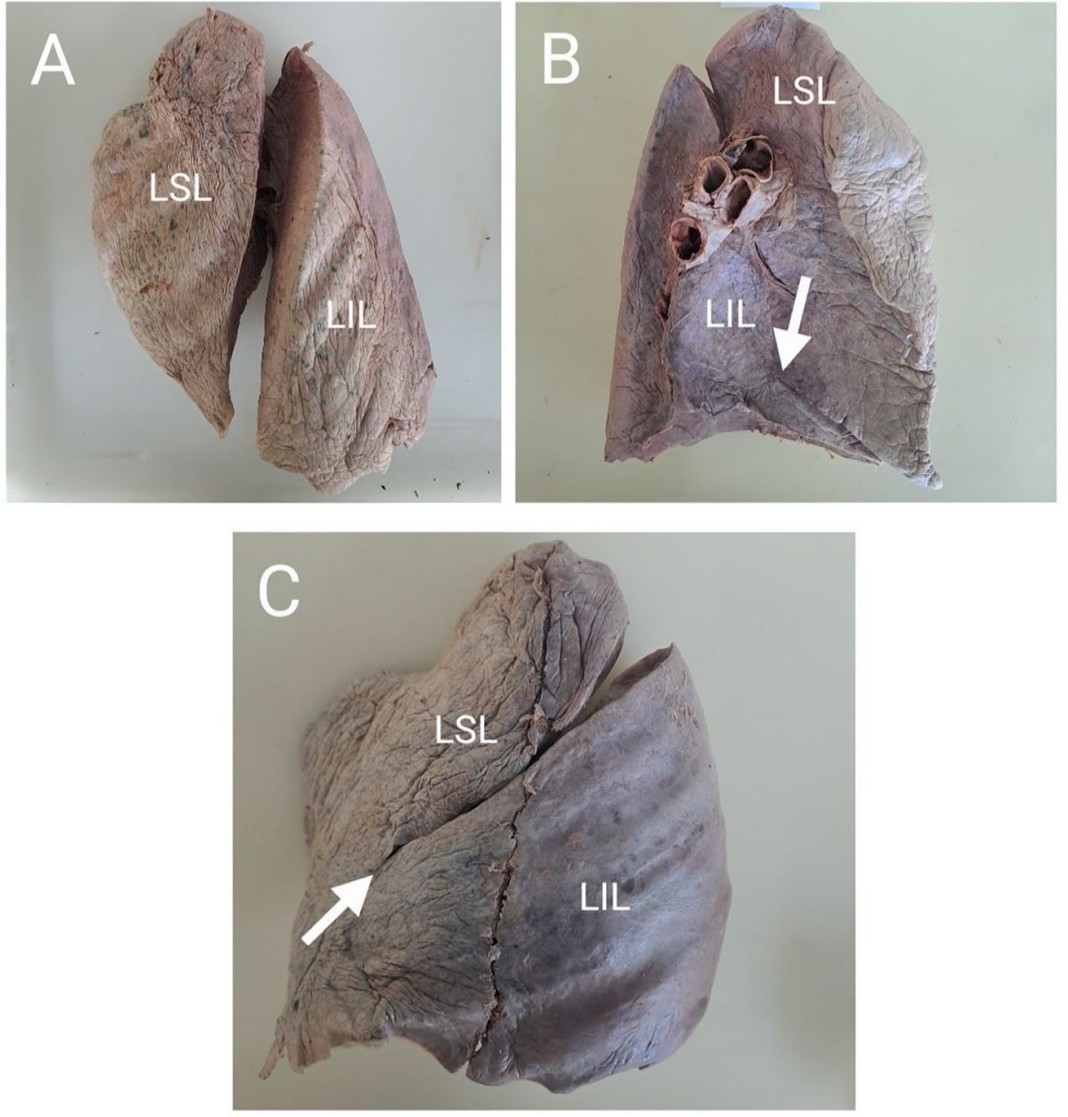
Morphological variations of the left lung. (**A**) Grade I oblique fissure separating the LSL and LIL completely. (**B**) Mediastinal surface showing Grade II oblique fissure that is partially incomplete. The oblique fissure reaches the hilum on the superior part but not on the inferior side, as indicated by the white arrowhead. (**C**) Grade III oblique fissure. The oblique fissure is incomplete, ending at the area indicated by the white arrowhead. Abbreviations: LIL=left inferior lobe, LSL=left superior lobe

The number of lobes in the left and right lungs were also recorded in the study. In the right lungs, 84.4% (*n* = 38/45) showed the usual morphology of three lobes (Fig. 1A), 6.4% (*n* = 2/45) had two lobes due an incomplete horizontal fissure, 6.7% (*n* = 3/45) had four lobes due to the presence of accessory fissures and 2.2% (*n* = 1/45) had a single lobe due to absence of both the horizontal and oblique fissures. In the left lungs, 93.6% (*n* = 44/47) had the usual morphology of two lobes (Fig. 2A), 2.1% (*n* = 1/47) had a single lobe due to an incomplete oblique fissure while 4.3% (*n* = 2/47) had three lobes due to the presence of accessory fissures. Figure 1 shows some of the anatomical variations found at the right lungs of different individuals.

Figure 2 shows some of the variant morphology at the left lungs of different individuals.

**Fig. 3:**
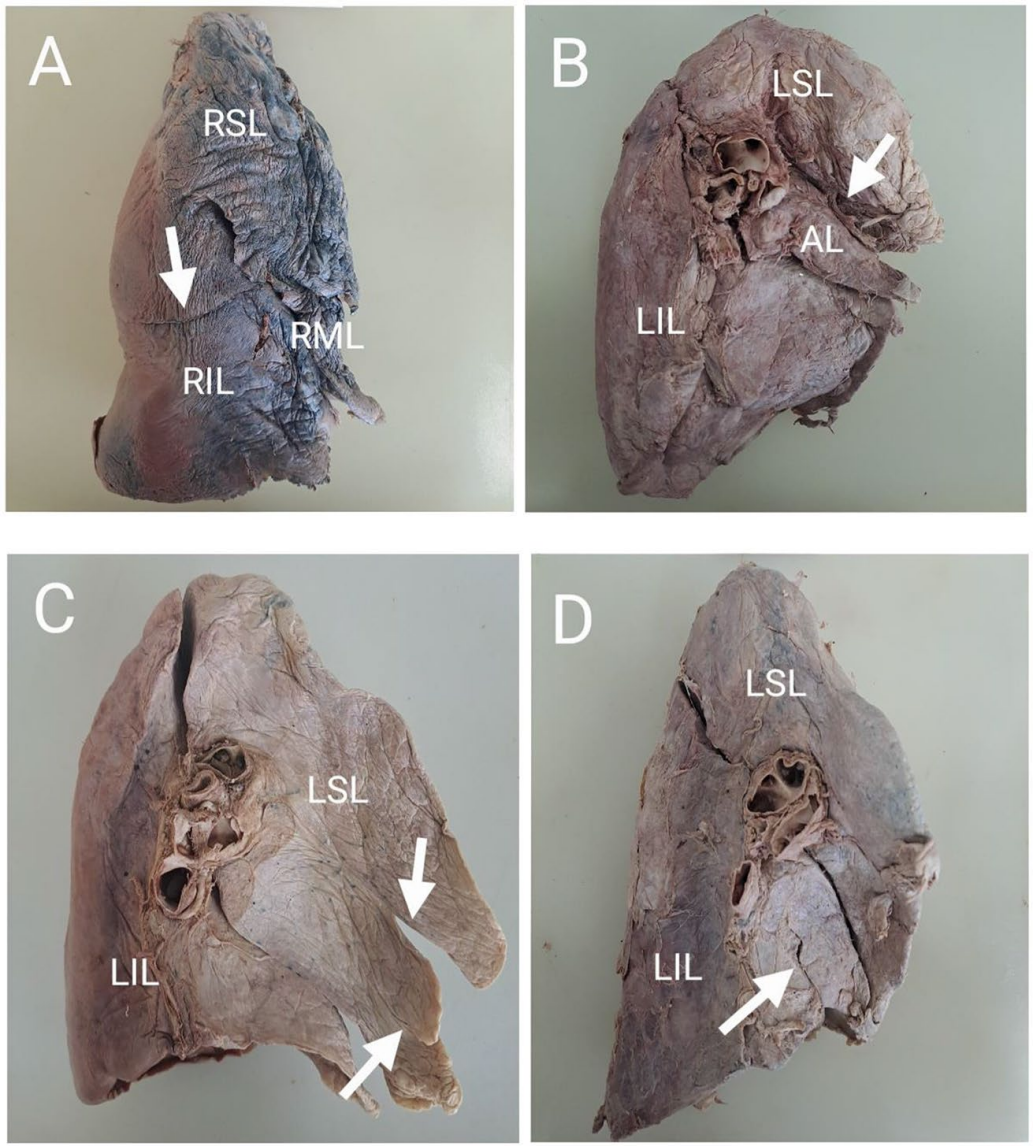
Accessory fissures and lobes found in both the right and left lungs of different individuals. (**A**) Right lung showing a superior accessory fissure, indicated by the white arrow, separating the RIL into two lobes thus resulting in four lobes. (**B**) Mediastinal surface of the left lung showing a complete oblique fissure along with a superior accessory fissure indicated by the white arrow, forming an accessory lobe. (**C**) The mediastinal surface of the left lung showing two left minor fissures, as indicated by the white arrows. (**D**) Mediastinal surface of the left lung showing an inferior accessory fissure as indicated by the white arrow. Abbreviations: RSL = right superior lobe, RIL = right inferior lobe, LSL = left superior lobe; LIL = left inferior lobe, AL = accessory lobe

Accessory fissures were observed in both the right and left lungs in the present study. Some of the accessory fissures are represented in Fig. 3. The superior accessory fissure (SAF) was found in 8.9% (*n* = 4/45) and 4.3% (*n* = 2/47) of the right and left lungs respectively. The inferior accessory fissure (IAF) was found in 2.2% (*n* = 1/45) and 2.1% (*n* = 1/47) of the right and left lungs respectively. The left minor fissure was found in 8.5% (*n* = 4/47) left lungs. One left lung had two left minor accessory fissures (Fig. 3C).

### Anatomical variations at the hilum

A total of 96 lungs were examined and among 90 lungs, the incidence of variations in the hilar pattern of 46 left and 45 right lungs was observed and recorded. Variations were seen in the number and arrangement of structures found at the hilum and presented in Tables 3 and 4.

Table 3 shows a summary of the frequency of variations in the number of structures at the hilum of both the left and right lungs.

**Table 3:** Frequency of variations in the number of structures at the hilum of both the right and left lungs

Hilar structure	Left lung *n* (%)					Right lung *n* (%)				
	One	Two	Three	Four	Five	One	Two	Three	Four	Five
Bronchus	42 (91.3%)	4 (8.7%)	0	0	0	15 (34.1%)	29 (65.9%)	0	0	0
Pulmonary artery	45 (97.8%)	1 (2.2%)	0	0	0	42 (95.5%)	2 (4.5%)	0	0	0
Pulmonary vein	11 (23.9%)	29 (63.0%)	5 (10.9%)	0	1 (2.2%)	6 (13.6%)	13 (29.5%)	21 (47.7%)	3 (6.8%)	1 (2.3%)

The hilar pattern of the left and right lungs showed varying percentages regarding the number of bronchi, pulmonary arteries, and pulmonary veins. Among these, the pulmonary vein was the most variable, ranging from a minimum of one to a maximum five pulmonary veins. Furthermore, the right lung showed the highest prevalence of unusual morphology of three pulmonary veins, seen in 47.7% (*n* = 21/45) lungs.

Table 4 shows the variations in the arrangement of the bronchi, pulmonary artery, and pulmonary vein at the hilum of both the left and right lungs.

**Table 4:** Frequency of variations in the arrangement of structures at the hilum of both the left and right lungs

Arrangement of structures at hilum	Right lung *n* (%)		Left lung *n* (%)	
	Usual	Variant	Usual	Variant
Superior to inferior	35 (79.5%)	9 (20.5%)	45 (97.8%)	1 (2.2%)
Anterior to posterior	33 (75.0%)	11 (25.0%)	36 (78.3%)	10 (21.7%)

In the right lungs, 79.5% (*n* = 35/45) and 75.0% (*n* = 33/46) lungs showed the usual pattern of structural arrangement from superior to inferior and anterior to posterior respectively (Fig. 5A). However, 20.5% (*n* = 9/45) of the right lungs deviated from the usual superior to inferior arrangement. Among the nine lungs, seven showed the following arrangement from superior to inferior: superior pulmonary vein, eparterial bronchus, pulmonary artery, hyparterial bronchus and inferior pulmonary vein (Fig. 5C). One lung displayed the following arrangement: pulmonary artery, bronchus and pulmonary vein. The remaining lung showed arrangement as superior pulmonary vein, eparterial bronchus, pulmonary artery, hyparterial bronchus and inferior pulmonary vein. Furthermore, 25.0% (*n* = 11/45) deviated from the usual anterior to posterior arrangement of structures by having the following pattern: pulmonary artery, pulmonary vein, and bronchus (Fig. 5E).

**Fig. 4:**
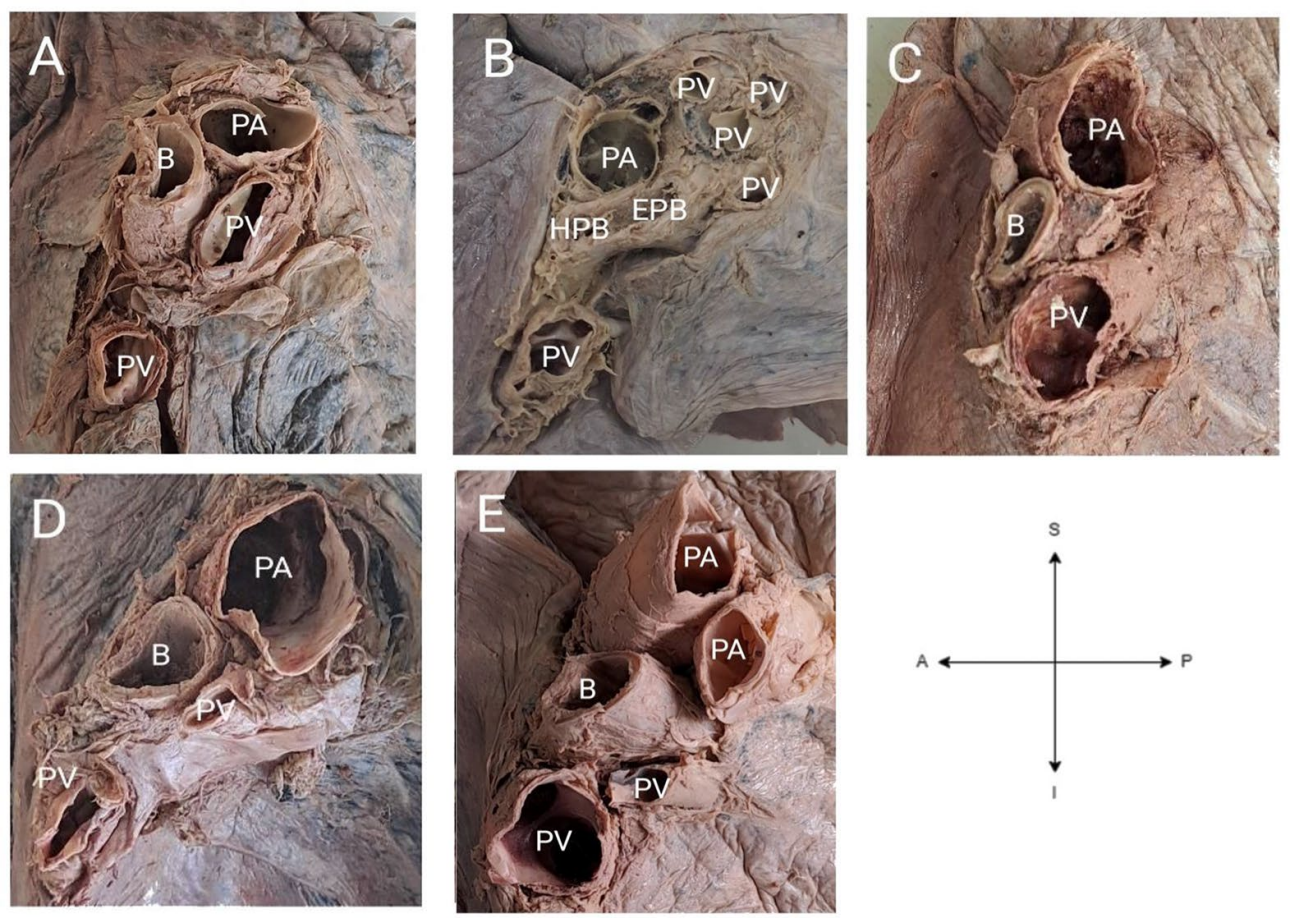
Anatomical variations of the left pulmonary hila. (**A**) Usual morphology of the number and arrangement of structures. (**B**) Hilum with two bronchi, one pulmonary artery and five pulmonary veins as well as a variant superior to inferior arrangement of structures (accessory pulmonary veins, pulmonary artery, eparterial bronchus, hyparterial bronchus and inferior pulmonary vein. (**C**) Hilum with one pulmonary artery, bronchus, and pulmonary vein. (**D**) Variant anterior-posterior arrangement of structures at hilum (pulmonary artery, pulmonary vein, and bronchus. (**E**) Hilum with a bronchus, two pulmonary veins and two pulmonary veins. Abbreviations: B= bronchus, PA=pulmonary artery, PV=pulmonary vein

In the left lungs, 97.8% (*n* = 45/46) and 78.3% (*n* = 36/46) lungs had the usual structural arrangement from superior to inferior and anterior to posterior respectively (Fig. 4A). Only 2.2% (*n* = 1/46) of the left lungs had a variant superior to inferior arrangement of structures as follows: accessory pulmonary veins, pulmonary artery, eparterial bronchus, hyparterial bronchus and inferior pulmonary vein (Fig. 4C). Ten lungs (21.7%) displayed a variant anterior to posterior arrangement of structures by having the following pattern: pulmonary artery, pulmonary vein and bronchus (Fig. 4E).

**Fig. 5:**
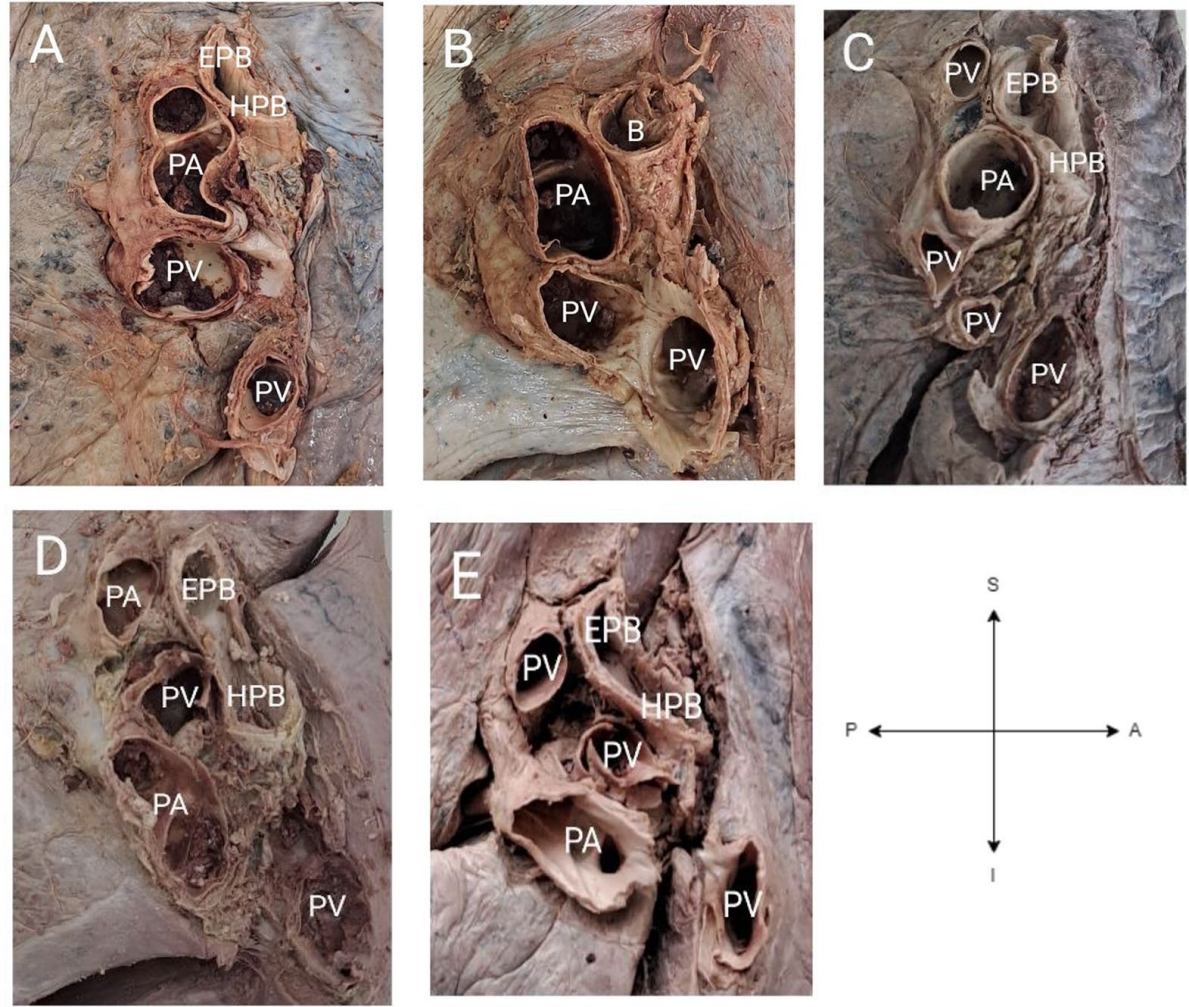
Anatomical variations of the right pulmonary hila. (**A**) Usual morphology of the number and arrangement of the structures. (**B**) Hilum with one bronchus, pulmonary artery and pulmonary vein. (**C**) Hilum with four pulmonary veins and variant superior-inferior arrangement of structures (pulmonary vein and eparterial bronchus, pulmonary artery, hyparterial bronchus, pulmonary veins). (**D**) Hilum with two pulmonary arteries, one pulmonary vein and the eparterial and hyparterial bronchus. (**E**) Hilum with three pulmonary veins and a variant anterior-posterior arrangement of structures (pulmonary artery, pulmonary veins, bronchus). Abbreviations: EPB= eparterial bronchus, HPA=hyparterial bronchus, PA=pulmonary artery, PV=pulmonary vein

Variant morphology of some hilum can be seen in Figs. 4 and 5.

Figure 4 shows some of the variant morphology in terms of the number and arrangement of structures at the hilum of the left lungs.

Figure 5 shows some of the variant morphology in terms of the number and arrangement of structures at the hilum of the right lungs of different individuals.

### Statistical analysis

It is important to note that all the fissure variations had expected counts of less than five, which does not satisfy the Chi-squared test criterion that requires each subcategory to have at least five counts. As a result, no meaningful conclusions could be drawn from the Chi-squared test. Additionally, normality tests were performed on the categorical data using a significance level of *p* < 0.05 and a 95% confidence interval. The results showed that all the data had p-values below 0.05, indicating no significant relationship between fissure variations and sex. For the numerical data, frequency analysis and normality tests were conducted using the same significance values to assess the relationship between hilar pattern variation and sex. The p-value for all the data was below 0.05, indicating that there was no significant relationship between hilar pattern variation and sex.

## Discussion

The lungs develop from a combination of two types of tissue, the endodermal and mesodermal tissues [42]. The endodermal tissue develops into the mucosal lining of the bronchi, as well as the epithelial cells of the alveoli. The mesodermal tissue develops into the vasculature of the lung along with the muscles and cartilage which support the bronchi [42]. Around the fourth week of embryonic development, the respiratory diverticulum (lung bud) grows caudally from the ventral wall of the foregut into the surrounding mesenchyme [17]. The lung bud will form the trachea and two (right and left) primary bronchial buds [25]. At the beginning of the fifth week, the right bronchial bud divides into three secondary bronchial buds while the left bronchial bud divides into two [16]. During the sixth week, the secondary bronchial buds repeatedly branch into tertiary bronchial buds eventually forming bronchopulmonary segments [17]. The bronchopulmonary segments are separated by gaps which eventually get obliterated except along two planes in the right lung, forming the horizontal and oblique fissure and one plane in the left lung which becomes the oblique fissure [23]. Along these fissures, the visceral pleura is reflected and envelops the individual lobes [25]. If non-obliteration of the gaps occurs, accessory fissures may persist while complete or partial obliteration will lead to absent or incomplete fissures [22].

Furthermore, pulmonary vessels initially develop within the mesenchyme by the process vasculogenesis [6]. Pulmonary arteries develop adjacent to the conducting airways, joining the angiogenic mesenchyme of the sixth aortic arch arteries [40]. Pulmonary veins develop from mediastinal mesenchyme in the dorsal mesocardium and become separated from the airways by the growth of alveoli and lymphatic vessels [40]. Deviations from the branching morphogenesis during embryonic and early fetal periods will result in variations in the hilum [6].

Various researchers have studied and reported on variant lung anatomy, as summarised in Tables 5 and 6, and 7. These findings are compared with those of the present study.

**Table 5:** Comparative prevalence of variations of oblique and horizontal fissures in both the left and right lungs

Author, year	Type of study	Region	Sample (*n*)	Right lung						Left lung		
				Horizontal fissure (%)			Oblique fissure (%)			Oblique fissure (%)		
				Complete	Incomplete	Absent	Complete	Incomplete	Absent	Complete	Incomplete	Absent
Gesase, [9]	cadaveric	Tanzania	102	-	7.84	-	-	0.98	-	-	10.78	-
Nene et al., [31]	cadaveric	India	50	78	8	14	92	6	2	88	12	0
Ghosh et al., [10]	cadaveric	India	82	26	26	47.8	79	19.56	2.17	79	13.88	5.55
Dutta et al., [5]	cadaveric	India	102	26.49	38.89	34.62	26.92	61.54	11.54	44	48	8
Jacob and Pillay [14]	cadaveric	India	48	10	83.4	6.6	46.6	50	3.4	61.1	38.9	0
George et al., [8]	cadaveric	India	138	61.55	35.38	3.07	96.93	3.07	0	84.94	15.06	0
Magadum et al., [22]	cadaveric	India	40	35	52.2	12.5	30	60	10	50	42.5	7.5
Mamatha et al., [23]	cadaveric	India	40	50	50	0	85	15	0	60	35	5
Kc et al., [17]	cadaveric	Nepal	50	52.18	34.78	13.04	69.57	30.43	0	48.15	51.85	0
Jacobs et al., [14]	cadaveric	India	96	15	70	15	49	51	0	34	62	4
Mutua et al., [30]	cadaveric	Kenya	48	47.3	42.1	10.5	63.1	36.8	0	65.6	34.4	0
West et al., [44]	cadaveric	United Kingdom	82	22.2	66.7	11.1	64.2	35.8	0	81.5	16.0	2.47
Ranaweeera et al., [35]	cadaveric	Sri Lankan	50	0	84.61	15.38	42.3	57.69	0	66.67	33.33	0
Mpolokeng et al., [26]	cadaveric	South Africa	82	40.2	39.0	7.3	57.3	29.3	-	57.3	29.3	1.2
Present study	cadaveric	South Africa	96	11.1	86.6	2.2	42.2	55.6	2.2	36.2	63.8	0

(- Indicates that the data was not provided)

Analysis of the studies in Table 5 reveal that incomplete fissures are common in lungs, with a higher prevalence seen in the right lung compared to the left lung.

Based on the findings from the current study, incomplete horizontal fissures had a higher prevalence of 86.6%. This is comparable to studies by Jacob and Pillay [13] and Ranaweeera et al. [35] where 83.4% and 84.6% of incomplete horizontal fissures was found, respectively. In contrast, Nene et al. [31] who examined 100 lungs, reported a relatively low prevalence of incomplete horizontal fissures, found in only 8% of the lung specimens. Similarly, a study carried out by Mutua et al. [30] contradicted the findings of the current study, reporting a lower incidence of incomplete horizontal fissures, present in 42.1% of the right lungs. A CT imaging study that analysed 560 cases by Manjunath et al. [24] reported an even lower incidence of 2.3% incomplete horizontal fissures while a study by Shinde and Patel [39] reported no incomplete horizontal fissures.

The present study recorded a low prevalence of absent horizontal fissures, with only 2.2% Grade IV horizontal fissures observed. This is comparatively similar to a study by George et al. [8] with a sample size of 138 lungs which reported absent horizontal fissures in 3.07% of right lungs. The highest prevalence was reported by Ghosh et al. [10], which had a relatively smaller sample size of 82 lungs, compared to the 96 lungs in the present study. In their study, Ghosh et al. [10] found absent horizontal fissure in 47.8% of lungs. Another study by Mamatha et al. [23] reported no absent horizontal fissures.

In the present study, the prevalence of right incomplete oblique fissures (Grade II and III) was found in 55.6% lungs, which is lower than that of incomplete horizontal fissures. This is comparable to studies by Dutta et al. [5] and Jacobs et al. [14] which reported 61.54% and 51% incomplete oblique fissures, respectively. Magadum et al. [22] deviated from this observation by having a higher incidence of incomplete oblique fissures compared to incomplete horizontal fissures. Gesase et al. [9], who investigated 102 lungs reported an even lower prevalence of incomplete right oblique fissures, present in 0.98% of the right lungs. According to Dutta et al. [5] a higher frequency of incomplete oblique fissures in the right lung could suggest an early onset of fusion of antenatal fissures, potentially proceeding further before birth, resulting in fusion along the base of the oblique fissure.

Similar to the horizontal fissures, the right oblique fissure was uncommonly absent. In this study, only 2.2% of Grade IV oblique fissures were observed in the right lungs, which is similar to findings by Nene et al. [31] which reported 2% of absent left oblique fissure. This is lower than what other studies found. Studies by Prakash et al. [33]; Dutta et al. [5] and Magadum et al. [22] reported absent right oblique fissures in 7.14%, 11.54% and 10% of the lungs, respectively. On the other hand, George et al. [8]; Mamatha et al. [23]; Wahane and Satpute [43]; West et al. [47] and Mutua et al. [29], found no absent oblique fissures.

In the current study, it was observed that incomplete oblique fissures were more prevalent in the left lungs when compared to the right lungs. Among the left lungs, 25.5% and 38.3% were Grade II and Grade III oblique fissures, respectively. This is similar to findings by Kc et al. [17] with a sample of 50 lungs, which reported incomplete oblique fissures in 51.85% of left lungs as opposed to 30.43% of right lungs.

Notably, none of the left lungs had an absent (Grade IV) oblique fissure in this study. This is consistent with what most studies reported (Table 5). On the contrary, Prakash et al. [33]; Dutta et al. [5]; West et al. [47] reported absent left oblique fissures in 10.7%, 8%, and 2.47% of the left lungs, respectively.

As reported by Li et al. [20], an incomplete interlobar fissure increases the surgical difficulty and the probability of prolonged air leaks. Patients that had incomplete interlobar fissures and went through a video-assisted thoracoscopic lobectomy had increased overall post-operative morbidity rate and were greatly associated with pneumothorax, pleural effusion, and atelectasis [19]. Huang et al. [12] reported that incomplete pulmonary fissures were greatly associated with post-operative complications such as prolonged length of chest tube drainage and hospital stay. Koster and Slebos [18] demonstrated that the possibility of collateral ventilation is high with incomplete pulmonary fissures. Furthermore, Okamoto et al. [32] reported that patients diagnosed with stage I non-small cell lung cancer (NSCLC), with incomplete fissures had higher occurrence of ipsilateral lung and lymph node metastases as well as poor prognosis compared to those with complete fissures. Similarly, Lee et al. [19] reported that patients with resected stage I adenocarcinoma with incomplete fissures had a reduced overall 5-year survival rate.

Supernumerary fissures, also known as accessory fissures, were identified in the present study. Anatomically, accessory fissures are described as clefts of varying depth lined by visceral pleura [17]. Literature describes accessory fissures as superior accessory fissure (SAF), inferior accessory fissure (IAF) also termed medial basal fissure and left minor fissure (LMF) also known as the left horizontal fissure [25]. The presence of accessory fissures at unusual locations in the lung result in anomalous lobes of the lung aerated by usual bronchus, which is commonly found in infants [26]. Accessory fissures found in the current study were compared to other studies summarised in Table 6.

**Table 6:** Incidence of accessory fissures in both the right and left lungs

Author	Right lung (%)		Left lung (%)		
	SAF	IAF	SAF	IAF	LMF
Nene et al., [31]	4	14	-	24	26
Quadros et al., [34]	8.33	5.55	-	5	17.5
Magadum et al., [22]	2.5	5	-	8	7.5
Kc et al., [17]	4.34	21.73	-	3.70	29.62
Wahengbam et al., [43]	7.14	21.43	2.70	43.24	29.73
Mutua et al., [30]	-	5.26	-	-	37.5
Present study	8.9	2.2	4.3	2.1	8.5

(- indicates that the data was not found)

In the present study, accessory fissures were present in both the left and right lungs. Among the right lungs, superior accessory fissures (SAF) were found in 8.9% of the lungs while they were found in 4.3% of the left lungs. These findings agree with studies by Quadros et al. [33], and Wahengbam et al. [44], who reported higher incidences of superior accessory fissures in the right lungs of 8.33% and 7.14%, respectively. A study by Godwin and Tarver [11] reported that superior accessory fissures (SAF) were present in 30% of the right lungs compared to the left lungs where an incidence ranging between 5% and 14% was observed. On the contrary, Mutua et al. [30] with a sample size of 96 lungs found no superior accessory fissures in both the left and right lungs.

Inferior accessory fissures (IAF) were observed in this study, with 2.2% found at the right lungs. This observation disagrees with majority of the studies which found higher incidences of IAF at the right lungs. Magadum et al. [22]; Quadros et al. [34] and Nene et al. [31] reported IAF in 5%, 5.5.% and 14% of the right lungs respectively. Kc et al. [17], and Wahengbam et al. [44] reported high incidence of IAF in 21.73% and 21.43% of the right lungs respectively. A CT imaging study by Manjunath et al. [24] reported inferior accessory fissures in 66.7% of the right lungs.

In the present study, 8.5% of the accessory fissures were left minor fissures (LMF) while 4.3% were left inferior accessory fissures (IAF). This incidence is lower compared to studies by Kc et al. [17] and Nene et al. [31] who reported high frequencies of LMF with 29.62% and 26% incidences. In contrast, a study conducted by Wahengbam et al. [44] deviated from this observation, reporting a higher prevalence (43.24%) of inferior accessory fissures (IAF) as opposed to left minor fissures (LMF).

A rare anatomical variation known as an azygous lobe, formed by the presence of an azygous fissure may be encountered in the right lung [13]. This variant is characterised by the partitioning of the right upper lobe due to the atypical course of the azygos vein, forming an additional lobe [25]. The prevalence of this variant lobe ranges from 0.4–1% in the general population [14]. Kc et al. [17], reported one case of an azygous lobe. However, this azygous lobe was not encountered in this study.

Presence of accessory fissures may form a barrier that will prevent the spread of infection, therefore causing marginated pneumonia [44]. Accessory fissures can be misdiagnosed as atelectasis, pleural scars, pleural effusion, or walls of bullae [21]. According to Dutta et al. [5] accessory fissures can change the typical pattern of lung collapse in patients with endobronchial lesions, making it difficult to diagnose a lesion and its extent.

The findings from the current study, along with comparisons to previous studies, reveal considerable variation in the prevalence of variant fissures and accessory fissures between and within populations. According to Mamatha et al. [23] these differences may be a result of genetic variations and environmental factors. Furthermore, Van der Molen et al. [41] who conducted a study on 9, 926 participants to determine environmental, clinical, and genetic determinants contributing to fissure completeness concluded that fissure completeness is genetically determined and independent on sex, age, and smoking status. Dogan et al. [3] states that lobar pattern abnormalities occur in conditions such as, polysplenia, and asplenia situs inversus viscerum, where these conditions cause defects in the determination of left-right asymmetry. Furthermore, Dogan et al. [3] states that accessory fissures may also alter the arrangement of structures at the hilum by dividing or creating additional compartments within the lung.

### Variations of the hilum structures

The present study observed variations at the hilum of the lungs, including differences in the number and arrangement of structures at both the left and right lungs. The observed findings were compared with those from studies summarised in Tables 7 and 8.

**Table 7:** Comparative prevalence of variations of the number of structures at the hilum of the right lung

Author, year	Region	Sample size (*n*)	Right lung structures (%)										
			1 Bronchus	2 Bronchi	3 Bronchi	1 Vein	2 Veins	3 Veins	4 Veins	5 Veins	1 Artery	2 Arteries	3 Arteries
George et al., [8]	India	138	-	98.46	1.53	-	63.07	32.30	-	-	29.23	67.69	3.07
Murlimanju et al., [28]	India	110	1.8	83.9	1.8	-	-	-	1.8	-	83.9	8.9	-
Jacobs et al., [14]	India	96	2	87	9	2	29	56	13	-	62	33	4
James et al., [15]	India	50	16	-	-	-	-	8	-	-	-	-	-
Saha and Srimani [36]	India	103	0	79.59	16.33	6.12	63.27	22.45	4.08	4.08	63.27	6.12	0
Amin, [17]	Egypt	40	-	76	24	-	62	-	-	-	71	29	38
Present study	South Africa	90	34.1	65.9	0	13.6	29.5	47.7	6.8	2.3	95.5	4.5	0

(- indicates that the data was not provided)

Table 8 shows the variations in the number of hilum structures observed in the left lung according to previous studies.

**Table 8:** Comparative prevalence of variations of the number of structures at the hilum of the left lungs

Author, year	Region	Sample size (*n*)	Left lung structures (%)										
			1 Bronchus	2 Bronchi	3 Bronchi	1 Vein	2 Veins	3 Veins	4 Veins	5 Veins	1 Artery	2 Arteries	3 Arteries
George et al., [8]	India	138	76.74	21.91	-	-	80.82	19.17	-	-	94.52	5.47	-
Murlimanju et al., [28]	India	110	51.8	35.2	7.4	-	51.8	1.8	-	-	51.8	5.5	-
Jacobs et al., [14]	India	96	43	49	8	4	75	17	2	2	83	17	-
James et al., [15]	India	50	-	-	-	-	-	-	-	-	-	-	-
Saha and Srimani [36]	India	103	53.70	37.04	9.26	9.26	68.52	14.81	3.70	3.70	62.96	27.78	9.26E
Amin, [15]	Egypt	40	79	21	-	-	63	37	-	-	84	16	-
Present study	South Africa	90	91.3	8.7	0	23.9	63.0	10.9	0	2.2	97.8	2.2	0

(- indicates that the data was not provided)

Variations at the hilum of the lungs were observed in the present study, with majority seen in the number of pulmonary veins of both the left and right lungs. These variations may lead to complications during surgical procedures, making it crucial for surgeons and clinicians to be aware of them [28].

Variant anatomy of the bronchi arising from the trachea or from the primary bronchi like the tracheal bronchus and accessory cardiac bronchus are uncommon. Nonetheless, knowing such congenital bronchial variations is vital for diagnosis and surgical interventions involving the trachea and bronchi [35]. In the present study, the usual morphology of two bronchi (eparterial and hyparterial) in the hilum of the right lung was observed in 65.9% of the specimens. This incidence is lower compared to studies by Murlimanju et al. [29]; George et al. [8]; Jacobs et al. [14], and Saha and Srimani [35] who reported two bronchi at the right hilum of 83.9%; 98%, 87% and 79.59% lungs respectively. The present study reported the highest frequency of one bronchus at the right hilum, seen in 34.1% lungs. Previous studies by Murlimanju et al. [29]; George et al. [8]; Jacobs et al., (2019); Saha and Srimani [35] and James et al. [15] with varying sample sizes of 110, 138, 96, 103 and 50 lungs, respectively, reported lower incidences of one bronchus ranging between 0- 16%. In the left hilum of this study, usual morphology of one bronchus was observed in 91.3% lungs while two bronchi were observed in 8.7% of the left hilum. This is not consistent with findings by George et al. [8] and Murlimanju et al. [29] who reported two bronchi in and 21.9% and 35.2% of the left lungs respectively.

Unforeseen bleeding originating from the pulmonary artery may be occasionally encountered by surgeons, thus knowledge of the pulmonary artery anatomy as well as their variations is vital to avoid complications [35]. The present study recorded a single pulmonary artery in 95.5% right lungs. This frequency is higher compared to a study by George et al. [8], which reported one pulmonary artery in 29.23% right lungs. Among the left lungs, 97.8% had one pulmonary artery which is similar to reports by George et al. [8], and Jacobs et al. [14] who reported an incidence of 94.52% and 83% respectively. The incidence of more than one pulmonary artery was low in this study. Two pulmonary arteries were observed in 4.5% and 2.2% of the right and left lungs respectively. Ganapathy et al. [6] with a sample size of 75 lungs reported a higher incidence of two pulmonary arteries, seen in 19.44% and 7.69% of the right and left lung. George e*t al.* [8] reported the highest incidence in the right lung with 67.69% of the specimens having two pulmonary arteries.

Understanding the anatomy and drainage pattern of pulmonary veins is important when performing procedures such as lobar resection [36]. Among the right lungs of this study, one pulmonary vein was observed in 13.6% of the lungs. This frequency is higher compared to the study by Saha and Srimani [36] who reported an incidence of 9.26%. The incidence of three pulmonary veins was found to be 47.7% in the right lungs of this study. This is similar to studies by Jacobs et al. [14] and George et al. [8] who reported three pulmonary veins in 56% and 32.30% right lungs, respectively. Four pulmonary veins were found in 6.8% of the right lungs while 2.3% had five. This is comparable to findings by Saha and Srimani [36] who reported four pulmonary veins in 4.08% of the right lungs and five pulmonary veins in 4.08% of the right lungs. Similarly, George et al. [8] reported more than three pulmonary veins in 4.61% of the right lungs. In the left lungs, three pulmonary veins were observed in 10.9% of the cases, which is comparable to findings by Jacobs et al. [14] which reported three pulmonary veins in 17% of the left lungs. Five pulmonary veins were observed in 2.2% of the left lungs in the present study, which is similar to findings by Jacobs et al. [14] which reported an incidence of 2%.

Variations in the arrangement of the structures was observed in the current study. Deviations from the usual arrangement of structures (superior-inferior and anterior-posterior) was seen in 45.5% right lungs and 23.9% left lungs. This observation does not agree with a study by Ganapathy et al. [6] which reported deviations from the usual arrangement of structures in 22.22% right lungs and 2.56% left lungs. In the present study, one right lung showed a variant superior-inferior arrangement of structures as pulmonary artery, bronchus, and pulmonary vein. A similar observation was reported by Ganapathy et al. [6] in six right lungs.

According to Murlimanju et al. [28], the presence of multiple pulmonary veins, pulmonary arteries, and multiple bronchi indicates a divergence in these structures prior to reaching the lung hilum. This divergence can be caused by deviations from the usual embryonic development.

Displacement of the hilum is a significant sign of pulmonary volume change (Saha and Srimani 2019). Wahengbam et al. [44] states that variant pulmonary veins are a dominant source of ectopic depolarisation, which can trigger atrial fibrillation in patients with paroxysmal atrial fibrillation. Additionally, Wahengbam et al. [44] stated that variations at the pulmonary artery increases the risk for vessel injury and procedure errors during pulmonary artery resection. Furthermore, variations at the hilum may resemble hilar masses which may potentially present difficulties for pulmonologists during clinical diagnosis [29].

## Conclusion

Variations in the completeness of fissures are a common occurrence, with the highest frequency of variations seen in incomplete fissures, specifically the horizontal fissure. The presence of accessory fissures was observed in both the left and right lungs, with a high prevalence of SAF and LMF. Additionally, variations in the number and arrangement of structures at the hilum are common, with the most frequent variation being the number of pulmonary veins at the right lung. It is important for surgeons and radiologist to be aware of these morphological variations as they may cause misdiagnosis and complications during surgical procedures.

## Data Availability

No datasets were generated or analysed during the current study.
